# The integration of physician associates into Lancashire and South Cumbria NHS Foundation Trust: a new role to address the challenge of physical health in mental health

**DOI:** 10.1192/bjb.2024.127

**Published:** 2025-02

**Authors:** Emma Glen

**Affiliations:** physician associate, Lancashire and South Cumbria Foundation Trust, UK. Email: Emma.Glen@lscft.nhs.uk

Physician associates are healthcare professionals who work as part of the multidisciplinary team (MDT) under the supervision of a named doctor (a consultant psychiatrist in mental health settings).^1^ They complete a 2-year postgraduate course which includes physical and mental health theory and clinical placements including mental health. In 2022, there were 3240 physician associates on the Managed Voluntary Register, with 4% of 454 census respondents working in psychiatry.^1^ Although this was a low number, it represented a doubling since the previous year. Lancashire and South Cumbria NHS Foundation Trust (LSCft) recruited their first physician associate in November 2021, and by 2024 numbers had increased to 14. These roles are in addition to foundation, core and specialty doctor posts. All LSCft physician associates work in in-patient settings, including forensic, psychiatric intensive care units, older adults, acute in-patients and rehabilitation. Their generalist training provides support with physical health, which enables resident doctors to focus on psychiatry. Early studies indicate that the physician associate role is highly valued in in-patient psychiatry.^2^

New healthcare roles present many challenges in their integration, and the physician associate role is no exception.^3^ There is a lack of knowledge and understanding of the role among both patients and colleagues, as well as some uncertainty about how best to utilise physician associates in psychiatry.^4^ However, the role has been extremely well received at LSCft, with consultants valuing the input of physician associates into the physical health management of their patients and the stability provided by their permanence within the MDT. LSCft has developed a range of strategies to aid the integration of physician associates, including:

- a physician associate forum;
- a physician associate peer support group, which has been particularly helpful in addressing physician associates' concerns around the emotive media coverage of their profession;
- a trust physician associate tutor, who is a consultant appointed to lead the physician associates;
- formal clinical supervision for 1 h per week with the named consultant;
- a physician associate handbook;
- funding for continuing professional development, which has been used to support attendance at medical and psychiatry conferences;
- funding for additional training, including clinical leadership and communication;
- a physician associate fellowship scheme, enabling successful applicants to complete continuous improvement projects, including physician associate supervision, oral health, diabetes simulation training and trauma-informed women's health;
- a physician associate representative delivering a presentation on their role to doctors joining the trust on rotation;
- a monthly physician associate education programme established by an ST clinical fellow, based on the Royal College of Psychiatrists’ national competence framework for physician associates working in mental health.^5^

These strategies showcase how physician associates can be successfully integrated within the in-patient psychiatry MDT, ensuring that they feel welcome and valued and allowing them to develop skills and competence in their roles while being safely supervised. LSCft and physician associates will work collaboratively in reviewing and implementing the Royal College of Psychiatrists’ physician associate guidelines for employers, supervisors and practitioners to ensure that physician associate practice is safe and that governance processes around their supervision and employment are clear. During a time in which in-patient physical and mental health acuity is changing, with potential under-recognition of how physical health needs contribute to this, as well as current workforce challenges, it is important to welcome innovative roles that can supplement existing ways of working. Using the methods outlined above may help other mental health trusts to recruit and retain physician associates in their workforce.
